# The Effect of the Difficulties Faced by Palliative Care Nurses on Perceived Quality of Palliative Care: A Cross‐Sectional Descriptive Study

**DOI:** 10.1111/scs.70000

**Published:** 2025-02-10

**Authors:** Uğur Öner, Zeliha Cengiz, Ahmet Erol

**Affiliations:** ^1^ Department of Fundamentals of Nursing, Faculty of Health Sciences Batman University Batman Turkey; ^2^ Department of Fundamentals of Nursing, Nursing Faculty Inonu University Malatya Turkey

**Keywords:** nurse, palliative care, palliative care difficulty, palliative care quality

## Abstract

**Aim:**

This study examined how the difficulties experienced by palliative care nurses while providing care affect the quality of palliative care perceived by nurses.

**Background:**

The increase in chronic diseases with the ageing population has brought about a greater need for palliative care. Palliative care nurses play a central role in improving patients' quality of life, while also facing emotional and physical challenges. The impact of these challenges on nurses' job satisfaction and the quality of care provided constitutes a critical area of research for understanding the effectiveness of palliative care services.

**Method:**

The study was conducted in a descriptive, cross‐sectional and correlational design with nurses working in palliative care services (*N* = 243) in Türkiye between October 2023 and March 2024. The data were collected through an introductory form, The Palliative Nursing Care Quality Scale and the Palliative Care Difficulties Scale. The relationship between the two variables was examined using Pearson correlation analysis, while the impact of palliative care challenges and education level on perceived palliative care quality was investigated through multiple linear regression analysis.

**Results:**

In this study, the data were normally distributed and it was found that the nurses' total mean score was 72.42 ± 8.26 (min: 18—max: 90) on the Palliative Nursing Care Quality Scale and 40.85 ± 10.50 (min: 15—max: 75) on the Palliative Care Difficulties Scale. Total mean palliative care quality score was found to be significantly higher among nurses with postgraduate degrees. Moreover, the palliative care difficulty scores were significantly higher for males, nurses with palliative experience of 1 year or less, and those who did not select the palliative care unit willingly. A negative correlation was observed between the nurses' quality of palliative care and their palliative care difficulties.

**Conclusion:**

It was seen that perceived quality of palliative care decreased as the difficulties encountered in palliative care increased. It is recommended to develop training programmes tailored for nurses and ensure continuous professional development to reduce the difficulties nurses face in palliative care and enhance the perceived quality of palliative care.

## 1 Introduction

The average life expectancy has increased with the developments in technology and healthcare services [1], which has led to a rise in the proportion of elderly population in the total population, leading to an increase in the incidence of chronic diseases. The increase in chronic diseases with the ageing population has brought about a greater need for palliative care [1]. The World Health Organisation (WHO) extimates that approximately 57 million individuals need palliative care every year around the world, which is projected to double by 2060 [2].

WHO defines palliative care as a comprehensive approach aimed at enhancing the quality of life for patients (both adults and children) and their families confronted with life‐threatening illnesses. This approach focuses on preventing and alleviating suffering through the early identification, thorough assessment, and effective management of pain and other challenges, including physical, psychosocial, and spiritual issues [2]. Palliative care is a significant part of nursing care, which requires nurses to employ their knowledge, attitudes and practical skills in combination [3]. Thus, it may become possible to provide a holistic and comprehensive care tailored to the patient's needs.

The aim of nursing care is to assist in meeting the patient's basic needs and ensure their comfort by considering the physical, psychological, cultural, intellectual, and social aspects of the patient [4]. Nurses should focus on providing quality care during their practice. Various definitions have been proposed regarding the concept of quality care, which is complex and multidimensional. In a qualitative study, nurses defined quality care as holistic patient care that encompasses meeting patients' needs, nurses' competence and empathy, optimal patient outcomes, and satisfaction [5]. İn order for nurses to be able to provide quality care, they need to possess the required knowledge and skills, be sensitive to the human and ethical aspects of the care, continuously develop their professional competence and provide services considering professional ethics [6]. Within the healthcare services, nurses can contribute to reducing hospital stay by ensuring cost‐efficiency. On the other hand, providing quality care could be difficulties at times. It could also be true for palliative care services [7]. The WHO reports that the primary difficulties faced by palliative care are the lack of trained human resources (healthcare professionals, home care providers and social workers, a multidisciplinary palliative care team etc.) and the insufficient understanding of palliative care among healthcare providers. They also mention the inadequacy of hospices and nurseries, poor physical health facilities, inefficient treatment methods for pain and other symptoms and the lack of a national palliative care strategy [2]. In addition, admissions to palliative services for other reasons than indications, families' concerns about not being able to care for their relative at home, the frequency of acute problems and legal issues are included among the challenges affecting the process of palliative care [8].

Palliative care nurses face various difficulties that affect their skills of providing qualitative care. These difficulties include insufficient knowledge of palliative care [9], emotional burden and ethical dilemmas [10], lack of confidence in providing palliative care [11] and factors that hinder nurses' development of their knowledge and skills in palliative care [12]. Moreover, nurses experience burnout due to the physical and mental difficulties brought about by their job [13], moral issues [14] and the difficulties of integrating all aspects of care for elderly patients with advanced dementia [15]. Similarly, organisational and systemic factors affect nurses' palliative care services negatively, as well. These factors include limited resources and lack of staff [16], time and resource constraints, and negative attitudes towards palliative care [17]. Considering the obstacles to a quality palliative care service, it could be suggested that nurses have difficulty when providing palliative care, and that particularly those who do not have a sufficient level of knowledge and experience of palliative care struggle to cope with these difficulties [8]. Difficulty in communication with patients and families [18], community coordination [19], alleviation symptoms and expert support [20] are the difficulties encountered in palliative care while providing palliative care as reported in the related literature.

In summary, it is crucial to deal with these multifaceted difficulties such as knowledge, emotional and ethical issues, burnout, moral difficulties and systemic barriers mentioned in relation to palliative care in order to empower nurses to provide high quality palliative care for patients and their families.

### 1.1 Aim of the Study

The study was conducted to examine the effect of the difficulties experienced by palliative care nurses while providing care on the quality of palliative care perceived by nurses.

### 1.2 Research Questions

1. What is the level of care quality perceived by palliative care nurses?
2. What is the level of difficulty faced by palliative care nurses?
3. What is the relationship between the difficulties encountered in palliative care and perceived quality of care?
4. How do the care difficulties encountered affect the quality of palliative care perceived by palliative care nurses?

## 2 Method

### 2.1 Design

The study is descriptive, cross‐sectional and correlational in nature.

### 2.2 Settings

Palliative care services are provided within the framework of healthcare quality standards in hospitals affiliated with the Ministry of Health, through multidisciplinary team collaboration. Palliative care is offered in palliative care units in hospitals across all 81 provinces of Türkiye. In accordance with healthcare service quality standards, palliative care clinics in Türkiye are required to have a responsible physician, with multidisciplinary teams engaging in coordinated planning. The facilities are also equipped with multi‐purpose rooms and comfortable patient rooms, which accommodate activities such as showering, family visits, and other engagements. Furthermore, the necessary equipment for emergency interventions is available. The care and treatment provided to patients are organised in a manner that ensures continuous and uninterrupted care.

### 2.3 Study Population and Sample

The population of the study consisted of nurses (*N* = 243) working in the palliative care services of all hospitals in XXX between October 2023 and March 2024. The sample size was calculated using the unknown population type method with Epi Info software (version 7.2.5.0). The sample from the unknown population comprised 243 nurses with 50% frequency, 5% margin of error (alpha:0.05) and 90% confidence interval.

Nurses who had been working in palliative care services of hospitals affiliated to the Ministry of Health for at least 1 year and who volunteered to participate in the study were included in the study. Nurses who had been working in palliative care for less than 1 year and nurses who were on leave such as maternity and nursing leave and who did not want to participate in the study were excluded from the study.

### 2.4 Data Collection Tools

The data were collected using an introductory form, The Palliative Nursing Care Quality Scale and The Palliative Care Difficulties Scale.

### 2.5 The Introductory Form

Developed by the researchers, the form consists of 7 questions asking about the palliative care nurses' age, gender, educational background, professional experience, experience in the palliative care unit, willingness to work in the palliative care clinic and previous training on palliative care.

### 2.6 The Palliative Nursing Care Quality Scale

The Palliative Nursing Care Quality Scale was developed by Zulueta‐Egea and colleagues in 2020 [21] and the Turkish validity and reliability study was conducted by Mollaoğlu and Boy in 2023 [22]. The scale comprises items that address the quality of care from a holistic perspective. It evaluates nursing professionals' perceptions of key aspects of palliative nursing care, including symptom management and relief, support for family members and/or caregivers, therapeutic relationships with patients and families, spiritual support, and continuity of care. The scale consists of 18 items and single dimension using 5‐point likert type. Possible scores to be obtained on the scale range between 18 and 90. Higher scores indicate good quality palliative care. While the Cronbach's Alpha value was 0.92 for the original scale, it was found as 0.89 in the present study.

### 2.7 The Palliative Care Difficulties Scale

The Palliative Care Difficulties Scale was developed by Nakazawa and colleagues in 2010 [23]. The Turkish validity and reliability study was carried out by Kudubes‐Akdeniz in 2019 [24]. The 15‐item tool describing the difficulties of palliative care is a 5‐Likert type scale. The scale consists of 5 subscales as “communication in multidisciplinary teams” (items 1–3), “communication with patient and family” (items 4–6), “expert support” (items 7–9), “alleviating symptoms” (items 10–12) and “community coordination” (items 13–15). Minimum and maximum scores that can be obtained from the scale are 15 and 75, respectively. Higher scores reveal increased difficulties faced by the individuals providing palliative care. The Cronbach's Alpha value of the original scale was 0.81, while it was 0.92 in the Turkish validity‐reliability study. In the present study, the Cronbach's Alpha value was found as 0.90.

### 2.8 Data Collection

The data were collected online via Google Forms. The first section of the Google Form provided information on the study and a consent box was added for voluntary participation. Those who agreed to the consent box were included in the study. Participants were sent a one‐time link. The study made adjustments to allow people to complete the surveys using Google Forms without revealing their name or personal information. These adjustments included “disabling the option to collect email addresses, removing the requirement to sign in with a Google account, not collecting indirect identifiers such as IP addresses, and selecting Allow multiple responses in the Google Forms settings”. Therefore, with these adjustments, direct identifiers such as name, email address, phone number, IP address were not collected during the survey. The online questionnaire, created using Google Forms, was shared with 394 palliative care nurses working in the palliative care clinics of hospitals affiliated with the Ministry of Health via the WhatsApp application. The convenience sampling method used to reach the sample is a fast and cost‐effective way of data collection, allowing access to a larger population. The study was completed with 243 nurses, and the response rate was determined to be 62%.

### 2.9 Data Analysis

The data were analysed using the SPSS 25.0 software. Descriptive statistics, reliability assessments, and tests for normality assumptions were conducted on the data. The normality assumption was assessed by examining the skewness and kurtosis values, following the ±2 guideline proposed by George and Mallery [25] and Tabachnick et al. [26]. Given that the skewness and kurtosis values were within the recommended range, it was concluded that the data satisfied the normal distribution assumption (Table 4). For the analysis of the data, independent samples *t*‐tests, ANOVA, Pearson correlation, and multiple linear regression analyses were performed. A significance level of *p* < 0.05 was considered acceptable for all statistical tests.

### 2.10 Ethical Considerations

Prior to initiating the study, ethical approval was obtained from İnönü University Health Sciences Non‐interventional Research Ethics Committee (Number: 2023/4948 and Date: 05.09.2023). In addition, nurses' consent was collected using an informed consent form via Google Forms.

## 3 Results

### 3.1 Participants Results

Sociodemographic characteristics of the nurses working in palliative care units are presented in Table 1. Accordingly, the mean age of the nurses is 31.91 ± 4.79, 78.2% are under 35 years‐old, 63.8% are males, 75.7% hold a bachelor's degree, 47.7% have 6–10 years of work experience, 26.3% have been working in the palliative care unit for 5 years or longer, 72% selected palliative care willingly, and 50.2% did not receive training on palliative care (Table 1).

**TABLE 1:** Sociodemographic characteristics of the nurses.

Demographic characteristics		
Age (mean±SD)	31.91 ± 4.79	(Min–Max: 23–48)
	*n*	%
Age		
Under 35 years‐old	190	78.2
36 years and older	53	21.8
Gender		
Female	88	36.2
Male	155	63.8
Educational background		
High school graduate	8	3.3
Associate degree	18	7.4
Bachelor's degree	184	75.7
Postgraduate degree	33	13.6
Professional experience		
1–5 years	85	35.0
6–10 years	116	47.7
11–20 years	38	15.6
21 years and over	4	1.6
Experience in the palliative care unit		
1 year	48	19.8
2 years	32	13.2
3 years	63	25.9
4 years	36	14.8
5 years and over	64	26.3
Willingness to choose palliative care		
Yes	175	72.0
No	68	28.0
Receiving education on palliative care		
Yes	121	49.8
No	122	50.2

### 3.2 Descriptive Results

The comparison of total mean scores on the palliative care quality scale according to the nurses' sociodemographic characteristics is given in Table 2. Nurses who have a postgraduate degree were found to have a significantly higher palliative care quality score compared to the other groups (*F* = 4.866; *p* < 0.03). The palliative care quality score of the nurses with a professional experience of 21 years and over was found to be lower than the other groups although the difference was not statistically significant. While the palliative care quality scale total mean scores of nurses who are males, have an experience of 5 years and over, selected palliative care willingly, and received palliative care training are relatively high, the difference between groups is not statistically significant (Table 2).

**TABLE 2:** Comparison of palliative care quality scale scores according to sociodemographic characteristics of nurses.

Demographic characteristics	Palliative care quality scale Mean ± SS	Statistical evaluation
Age		
Under 35 years‐old	72.34 ± 8.53	*t* = 0.266
36 years and older	72.69 ± 7.25	*p* = 0.78
Gender		
Female	71.57 ± 8.99	*t* = 1.266
Male	72.90 ± 7.80	*p* = 0.23
Educational background		
High school graduate	71.75 ± 12.14	***F* = 4.866** ***p* = 0.03**
Associate degree	73.11 ± 9.19	
Bachelor's degree	71.51 ± 8.24	
Postgraduate degree	77.30 ± 4.66	
Professional experience		
1–5 years	71.24 ± 8.44	*F* = 2.222 *p* = 0.08
6–10 years	73.22 ± 8.09	
11–20 years	73.39 ± 7.92	
21 years and over	65.00 ± 8.48	
Experience in the palliative care unit		
1 year	71.89 ± 10.55	*F* = 0.388 *p* = 0.81
2 years	71.56 ± 8.26	
3 years	72.34 ± 7.26	
4 years	72.19 ± 7.62	
5 years and over	73.45 ± 7.72	
Willingness to choose palliative care		
Yes	72.75 ± 8.09	*t* = 1.000
No	71.57 ± 8.70	*p* = 0.33
Receiving education on palliative care		
Yes	73.39 ± 7.91	*t* = 1.837
No	71.45 ± 8.51	*p* = 0.06

*Note:* Bold values indicate that the result is statistically significant (*p* < 0.05).

### 3.3 Comparative Results

The comparison of the total and subscale scores of the Palliative Care Difficulties Scale according to the sociodemographic characteristics of nurses working in palliative care units is presented in Table 3. It was seen that the total mean score on the palliative care difficulties scale and the scores of expert support and community coordination subscales were statistically significant in terms of gender, with male nurses having a higher mean score than female nurses (*p* < 0.05). It was determined that the scores of educational background and communication with the patient and families were statistically significant, and high school graduates had a higher score than the others (*p* < 0.05). Total mean score on the palliative care difficulty scale and the mean scores on the communication with the patient and families and alleviating symptoms subscales were statistically significant in terms of palliative care nurses' experience, and nurses with a 1‐year of working experience were seen to have significantly higher mean scores compared to the other durations of experience. The difference between the nurses' total mean scores on the palliative care difficulty scale in terms of selecting palliative care willingly was statistically significant. Mean difficulty score of the nurses who were unwilling to work at palliative care units was higher than those who were willing (Table 3).

**TABLE 3:** Comparison of total and subscale scores of palliative care difficulties scale according to sociodemographic characteristics of nurses.

	Communication in multidisciplinary teams	Communication with the patient and family	Expert support	Alleviating symptoms	Community coordination	Palliative care difficulties scale total score
Demographic characteristics	Mean ± SS	Mean ± SS	Mean ± SS	Mean ± SS	Mean ± SS	Mean ± SS
Age						
Under 35 years‐old	8.97 ± 2.64	8.93 ± 2.56	7.61 ± 2.38	7.71 ± 3.24	7.99 ± 2.69	41.22 ± 10.44
36 years and older	8.41 ± 2.36	8.69 ± 2.72	8.11 ± 2.47	6.33 ± 3.22	7.96 ± 2.20	39.52 ± 10.72
Significance	*t* = 1.401 *p* = 0.16	*t* = 0.559 *p* = 0.56	*t* = 1.345 *p* = 0.18	*t* = 2.734 *p* = 0.007	*t* = 0.108 *p* = 0.93	*t* = 1.041 *p* = 0.30
Gender						
Female	8.57 ± 2.36	8.80 ± 2.55	7.15 ± 2.14	6.95 ± 3.35	7.54 ± 2.52	39.04 ± 9.78
Male	9.02 ± 2.71	8.92 ± 2.62	8.03 ± 2.49	7.67 ± 3.22	8.23 ± 2.60	41.88 ± 10.79
Significance	*t* = 1.253 *p* = 0.21	*t* = 0.339 *p* = 0.73	***t* = 2.892** ***p* = 0.006**	*t* = 1.622 *p* = 0.10	***t* = 2.037** ***p* = 0.045**	***t* = 2.093** ***p* = 0.038**
Educational background						
High school graduate	9.25 ± 2.60	11.12 ± 2.35	7.87 ± 3.64	7.12 ± 2.94	7.87 ± 3.31	43.25 ± 11.01
Associate degree	9.27 ± 2.08	9.77 ± 2.60	7.88 ± 2.60	8.38 ± 3.68	8.61 ± 2.17	43.94 ± 9.48
Bachelor's degree	8.70 ± 2.60	8.80 ± 2.56	7.80 ± 2.38	7.47 ± 3.28	8.00 ± 2.63	40.84 ± 10.69
Postgraduate degree	9.18 ± 2.85	8.24 ± 2.52	7.09 ± 2.09	6.65 ± 3.02	7.57 ± 2.34	38.66 ± 9.74
Significance	*F* = 0.515 *p* = 0.47	***F* = 3**.**526** ***p* = 0.02**	*F* = 0.874 *p* = 0.62	*F* = 1.293 *p* = 0.29	*F* = 0.630 *p* = 0.39	*F* = 1.136 *p* = 0.16
Professional experience						
1–5 years	8.50 ± 2.45	9.07 ± 2.50	7.44 ± 2.47	7.62 ± 3.44	7.92 ± 2.87	40.57 ± 11.11
6–10 years	8.83 ± 2.76	8.85 ± 2.59	7.71 ± 2.24	7.62 ± 3.44	7.80 ± 2.48	39.97 ± 10.48
11–20 years	9.63 ± 2.31	9.05 ± 2.56	8.13 ± 2.55	7.02 ± 3.24	8.50 ± 2.22	43.57 ± 9.14
21 years and over	9.50 ± 1.73	11.50 ± 2.38	9.75 ± 3.77	6.00 ± 1.00	9.75 ± 1.50	46.50 ± 5.74
Significance	*F* = 1.747 *p* = 0.15	*F* = 2.059 *p* = 0.10	*F* = 1.694 *p* = 16	*F* = 1.764 *p* = 0.15	*F* = 1.331 *p* = 0.26	*F* = 1.538 *p* = 0.20
Experience in the palliative care unit						
1 year	9.29 ± 2.28	10.08 ± 2.22	7.91 ± 2.29	8.77 ± 2.90	8.20 ± 2.66	44.27 ± 943
2 years	8.12 ± 2.52	8.31 ± 2.58	7.31 ± 2.27	6.81 ± 3.39	8.00 ± 2.71	38.10 ± 10.54
3 years	8.80 ± 2.90	8.30 ± 2.38	7.46 ± 2.46	6.92 ± 3.46	8.07 ± 2.56	39.57 ± 10.55
4 years	8.40 ± 2.28	8.63 ± 3.16	7.69 ± 2.24	6.19 ± 3.06	7.36 ± 2.46	38.36 ± 11.06
5 years and over	9.15 ± 2.65	8.96 ± 2.47	8.04 ± 2.59	7.85 ± 3.09	8.07 ± 2.59	42.10 ± 10.33
Significance	*F* = 1.398 *p* = 0.24	***F* = 4.030** ***p* = 0.01**	*F* = 0.784 *p* = 0.53	***F* = 4.442** ***p* = 0.01**	*F* = 0.649 *p* = 0.60	***F* = 2.693** ***p* = 0.03**
Willingness to choose palliative care						
Yes	8.69 ± 2.64	8.78 ± 2.60	7.57 ± 2.41	7.80 ± 2.60	7.82 ± 3.15	39.88 ± 10.48
No	9.27 ± 2.44	9.11 ± 2.58	8.08 ± 2.36	8.47 ± 2.50	8.40 ± 2.50	43.36 ± 10.20
Significance	*t* = 1.590 *p* = 0.11	*t* = 0.88 *p* = 0.37	*t* = 1.488 *p* = 0.13	*t* = 1.820 *p* = 0.07	*t* = 1.854 *p* = 0.06	***t* = 2.344** ***p* = 0.019**
Receiving education on palliative care						
Yes	9.00 ± 2.61	9.38 ± 2.68	7.81 ± 2.57	7.61 ± 3.08	8.02 ± 2.75	42.04 ± 10.67
No	8.71.± 2.58	8.38 ± 2.41	7.62 ± 2.24	7.20 ± 3.47	7.74 ± 2.40	39.67 ± 10.67
Significance	*t* = 0.86 *p* = 0.39	*t* = 3.036 p = 0.003	*t* = 0.631 *p* = 0.52	*t* = 0.984 *p* = 0.32	*t* = 1.464 *p* = 0.14	*t* = 1.771 *p* = 0.07

*Note:* Bold values indicate that the result is statistically significant (*p* < 0.05).

The average scores of nurses' palliative care difficulty and its subdimensions, along with the Palliative Care Quality Scale, are presented in Table 4. Upon reviewing Table 4, it was found that the total average score of the Palliative Care Quality Scale was 72.42 ± 8.26, while the total average score of the Nurses' Palliative Care Difficulty Scale was 40.85 ± 10.50. The subscale means for communication in multidisciplinary teams were 8.85 ± 2.59, communication with the patient and family were 8.88 ± 2.69, expert support was 7.72 ± 2.40, alleviating symptoms was 7.41 ± 3.28, and Community coordination was 7.98 ± 2.59. A positive, low‐level correlation was found between the total scale scores of the Palliative Care Quality Scale and the Nurses' Palliative Care Difficulty Scale (*r* = 0.143, *p* = 0.001). A positive, moderate‐level correlation was found between the Palliative Care Quality Scale and the communication in multidisciplinary teams subscale scores (*r* = 0.50, *p* = 0.001), and a positive, low‐level correlation was found between the Palliative Care Quality Scale and the expert support subscale scores (*r* = 0.159, *p* = 0.001) (Table 4).

**TABLE 4:** The relationship between the total scores and subscales of the palliative care quality scale and palliative care difficulties scale.

Variables	Mean	SD	Skew.	Kurt.		1	2	3	4	5	6	7
Palliative care quality scale	72.42	8.26	−0.15	−0.13	Pearson correlation	1						
					Sig. (2‐tailed)	—						
The palliative care difficulties scale	40.85	10. 50	0.45	−0.25	Pearson correlation	−0.143	1					
					Sig. (2‐tailed)	0.02	—					
Communication in multidisciplinary teams subscale	8.85	2.59	0.12	−0.65	Pearson correlation	−0.50	0.715	1				
					Sig. (2‐tailed)	0.46	0.01	—				
Communication with the patient and family subscale	8.88	2.69	0.37	−0.69	Pearson correlation	−0.104	0.776	0.470	1			
					Sig. (2‐tailed)	0.10	0.01	0.01	—			
Expert support subscale	7.72	2.40	0.75	−0.11	Pearson correlation	−0.159	0.765	0.412	0.489	1		
					Sig. (2‐tailed)	0.01	0.01	0.01	0.01	—		
Alleviating symptoms subscale	7.41	3.28	0.26	−0.98	Pearson correlation	−0.084.	0.883	0.474	0.581	0.515	1	
					Sig. (2‐tailed)	0.19	0.01	0.01	0.01	0.01	—	
Community coordination subscale	7.98	2.59	0.55	−0.34	Pearson correlation	−0.172	0.793	0.435	0.477	0.618	0.574	1
					Sig. (2‐tailed)	0.07	0.01	0.01	0.01	0.01	0.01	—

The multiple linear regression results regarding the effect of nurses' the difficulties encountered in palliative care and educational background on the palliative care quality perceived by nurses are given in Table 5. According to Table 5, palliative care difficulty and educational background explains 7.4% of the palliative care quality perceived by nurses (*R* ^2^ = 0.074, *β* −0.130). Among the sociodemographic variables, education level was the only factor that demonstrated a statistically significant difference in the mean scores for perceived quality of palliative care. Consequently, education level was coded as a dummy variable and included in the multiple linear regression model. The results suggest that, when undergraduate graduates serve as the reference group, postgraduate graduates have a significant impact on the perceived quality of palliative care (*β* = 0.231; *p* < 0.05) (Table 5).

**TABLE 5:** The result of the regression analysis of the effect of nurses' palliative care difficulties and educational background on palliative care quality.

	Depend variable		Coefficients					
Independent variable	Palliative care quality	Unstandized *β*	Std. error	Standized coefficients *β*	*t*	*p*	*R*	*R* ^2^
	Constant variable	75.678	2.102		35.999	0.000	**0.272**	**0.074**
Palliative Care Difficulties Scale		−0.102	0.049	−0.130	−2.065	**0.040**		
High school graduate^a^		0.485	2.898	0.010	0.167	0.867		
Associate degree^a^		1.917	1.986	0.061	0.965	0.335		
Postgraduate degree^a^		5.570	1.519	0.231	3.666	**0.000**		

*Note:* Bold values indicate that the result is statistically significant (*p* < 0.05).

^a^ Reference: Bachelor's degree.

## 4 Discussion

This study examines the effect of the difficulties experienced by palliative care nurses while providing care on the quality of palliative care perceived by nurses. The palliative care quality perceived by nurses was found to be high in the present study. Similarly, the study conducted by Zulute Egea and colleagues stated that the quality of palliative care perceived by nurses was high, while Zhang and colleagues reported a low quality of palliative care perceived by nurses [7, 27]. The higher quality of palliative care found in the present study could have been caused by the participants who received palliative care training, hold a postgraduate degree and chose palliative care willingly. A review of the literature evaluating the quality of care provided by nurses through various scales reveals studies reporting care quality at moderate [28, 29] and high levels [30, 31].

It is noteworthy that the mean scores of the nurses with a postgraduate degree are significantly high in the present study (Tables 2 and 5). Nursing education is an applied profession based on knowledge and skills. Considering that postgraduate education potentially improves nurses' knowledge and skills further, it could be suggested that nurses' knowledge and skills increase parallel with their education level, which in turn enhances the quality of palliative care perceived by nurses. It can be stated that the quality of palliative care perceived by nurses tends to increase as nurses' age, gender, professional experience, and duration of palliative care experience increase, and if nurses received palliative care training. Although the data are not statistically significant, the increased scores are worth considering for guiding for future research. An interesting finding is that the quality of palliative care perceived by nurses, which increases as professional experience increases, tends to decrease in later years of the profession. In the present study, it is seen that the nurses who have professional experience of 21 years and over have the lowest palliative care quality perceived by nurses compared to the other groups. Although professional experience positively affects problem solving, communication and clinical decision‐making skills, nursing profession is physically, mentally and emotionally demanding and exhausting. Thus, we believe, the palliative care quality perceived by nurses' decreases after a certain period. It is suggested that male nurses in palliative care services may enhance the palliative care quality perceived by nurses due to their psychological resilience and the increased time spent with patients during activities such as repositioning and providing direct care. Zhang and colleagues reported the factors affecting the palliative care quality perceived by nurses as age and receiving education on palliative care [27]. In the study conducted by Altarawneh and colleagues, the palliative care quality perceived by nurses scores were found significantly higher among nurses who were males, had received palliative care training and chose the palliative care unit willingly [3]. Zulute Egea and colleagues carried out a qualitative study using the palliative care quality scale and concluded that the subscales affecting the palliative care quality perceived by nurses were symptom control and alleviation, therapeutic relationship and spiritual support [7].

The present study, the difficulties encountered in palliative care score was found to be 40.85 (Table 4). Similarly, in studies comparable to ours, the palliative care difficulty scores were 42.3 in the study by Atakoğlu‐Yılmaz et al. for nurses, 44.3 in the study by An R. et al., 38.94 in the study by Nguyen et al., and 44.9 in the study by Ünver et al. for nursing students. In our study, among the subscales of palliative care difficulties, the highest score was found in the “communication with the patient and family” subscale (8.88), while the lowest score was in the “alleviating symptoms” subscale (7.41) (Table 4) [18, 19, 32, 33]. There are studies in the literature reporting that the highest palliative care difficulty belongs to the “communication with the patient and families” subscale [18, 20, 34, 35, 36, 37], similarly to the data of the present study. Researchers such as Atakoğlu et al., Toh et al., and Danielsen et al. have highlighted that elevated scores in the “communication with the patient and family” subscale are linked to sudden changes in the patient's condition, difficulties in conveying death notifications, and challenges encountered in clinical decision‐making [18, 35, 37]. Likewise, Ichikura's study underscores communication issues during the provision of psychological and physical care for the patient [20]. In Kobayashi's research, the lack of knowledge and skills was identified as the primary factor contributing to communication difficulties [36]. Furthermore, in the study by Okamoto et al., conducted with assistant physicians, high scores in the “communication with the patient and family” subscale were attributed to challenges in delivering death notifications and addressing ethical dilemmas in treatment decisions [34]. Unlike the present study, some studies found the highest palliative care difficulty scores on other subscales such as alleviating symptoms [20, 33, 38], expert support [34] and community coordination [19, 32]. High scores found on the communication with the patient and families subscale are associated with the difficulty when making explanations to the family, difficulties faced when the patient's condition deteriorates, fear of death among families, cultural differences, distrust in healthcare personnel, lack of knowledge of therapeutic communication techniques, difficulty in showing empathy and problems experienced during active listening.

In the present study, the difficulties encountered in palliative care scores were found to be significantly higher among male nurses, nurses with palliative care experience of 1 year and those who did not select the palliative care unit willingly. Some studies conducted previously report high the difficulties encountered in palliative care scores among females, but the results are not statistically significant [18, 19]. Additionally, a limited number of studies concerning the duration of palliative care experience support the findings of our study [32, 38]. Since the difficulties encountered in palliative care and complicated, requiring advanced care skills, effective communication with patients and their families and symptom management skills, it is evident that nurses with a clinical experience of as short as 1 year may have deficiencies in coping with difficulties and clinical practice, as well as adapting to palliative patients with low well‐being. None of the previous studies looked into whether nurses choose to work in palliative care units or not. This unique aspect of our study indicates that nurses can experience difficulty due to being forced to work in a field they do not want, feeling a lack of belonging to the clinic and the belief that they may not be happy working in the clinic.

In our study, although nurses who were under 35 years‐old, were high school graduates, those with a professional experience of 21 years and more and nurses who received palliative care training had higher scores of the difficulties encountered in palliative care, they were not statistically significant. Nguye and colleagues reported that older age, having a bachelor's level of education and receiving training on palliative care were associated with significantly higher the difficulties encountered in palliative care scores among nurses [32]. Atakoğlu‐Yılmaz and colleagues stated that nurses who completed bachelor's degree and did not receive palliative care training had significantly higher the difficulties encountered in palliative care scores [18]. Toh SW. and colleagues found that nurses with less clinical experience and lower education levels had higher and statistically significant communication difficulty scores [37]. The reason for the difficulties faced by nurses under 35 years‐old may be attributed to the fact that they can improve their conscious mind and abilities to cope with difficulties through experience, as they grow older. Higher the difficulties encountered in palliative care scores with increased professional experience is thought to result from heavy workload, burnoutand insufficient energy to provide holistic care and decreased consciousness and attitudes while providing care. High difficulty scores among nurses who received palliative care training, on the other hand, indicate that individual improvement of nurses is not sufficient for overcoming all the difficulties encountered in palliative care.

A negative and statistically low‐level relationship was found between the palliative care quality perceived by nurses scale and the difficulties encountered in palliative care in our study (Table 5). As the difficulty nurses experience while providing palliative care increases, the quality of care decreases, while the quality increases as difficulties decrease. Similarly, Matchim Y and colleagues found that the quality of care decreased as care difficulty scores increased [39]. The finding of a low‐level negative effect of palliative care challenges on the quality of care perceived by nurses in the regression analysis reflects the coexistence of a range of factors influencing care quality. The emotional, psychological, and physical challenges faced by nurses may negatively impact the perceived quality of care; however, this effect is not limited to challenges alone. Perceived care quality is also shaped by various other factors such as organisational elements, in‐house support systems, teamwork, nurses' education level, and professional experience. Additionally, the impact of challenges faced by nurses may vary depending on factors such as individual resilience, professional attitudes, and psychological support in the workplace. This indicates that the effect of challenges on perceived care quality is limited and complex [27, 29, 30]. When undergraduate graduates are considered as the reference group, it has been determined that postgraduate graduates have a significantly positive impact on the quality of palliative care (Table 5). To the best of our knowledge, there are no studies in the literature addressing this specific issue; therefore, our study provides a unique and innovative contribution in this regard. The higher scores of postgraduate graduates in perceived palliative care quality are generally attributed to their more detailed academic knowledge and clinical experience. Postgraduate education not only provides theoretical knowledge but also equips individuals with practical skills, allowing them to reach a higher level of professional competence that can enhance palliative care units.

### 4.1 Limitations of the Study

This study has some limitations. The results of the study are limited to the nurses who worked in the palliative care service and volunteered to participate in the study. Additionally, the data of the study rely on the nurses' verbal statements. Therefore, it is assumed that nurses responded to all questions sincerely and honestly. One of the limitations of this study is that the data were collected in an online based surveys. Self‐assessment methods may introduce social desirability biases. To mitigate this bias, the collected data were assured to anonymity of responses. Therefore, it is suggested that data collection be conducted through observational research to mitigate such biases. A limitation of our study is the predominance of male participants and the 90% confidence interval. A total of approximately 394 palliative care nurses were contacted, of whom 243 completed the questionnaire, and the response rate was determined to be 62%.

## 5 Conclusion and Recommendations

It was found that the palliative care quality perceived by nurses' palliative care was above average, while the difficulties encountered in palliative care level was below average. Nurses who hold a postgraduate degree were found to have significantly higher levels of palliative care quality perceived by nurses. A weak negative relationship was determined between the palliative care quality perceived by nurses and the difficulties encountered in palliative care of the nurses. The highest the difficulties encountered in palliative care score was obtained on the “communication with the patient and families” subscale. The factors affecting the difficulties encountered in palliative care were found to include gender, duration of palliative care experience, and willingness to choose the palliative care unit.

In order to improve the quality of palliative care perceived by nurses and to reduce the difficulties encountered, it is recommended to implement targeted training programmes that focus on developing basic skills such as active listening, empathy, helpfulness, compassion and effective communication among nurses. Furthermore, it is advised that regular interdisciplinary meetings be conducted between nurses and other members of the healthcare team to facilitate case management, promote collaboration, and ensure continuity of care. Incorporating palliative care courses into the undergraduate nursing curriculum will increase the desire and experience of nurses by giving them early exposure to palliative care. Therefore, it is recommended that courses related to palliative care should be included in the undergraduate curriculum. Institutions should identify the difficulties in providing palliative care and provide caregivers with opportunities to ensure quality palliative care. It would be useful for further research to conduct observational studies on palliative care nurses.

## Author Contributions

**Uğur Öner:** formal analysis, investigation, data collection, visualisation, writing – original draft, writing – review and editing, methodology. **Zeliha Cengiz:** formal analysis, investigation, validation, visualisation, writing – original draft, writing – review and editing. **Ahmet Erol:** formal analysis, investigation, validation, visualisation. All authors participated in the design of the study and in data analysis, and interpretation. All authors contributed to data collection and manuscript writing. All authors have read and approved the final version of the manuscript and agreed with the order of presentation of the authors.

## Ethics Statement

Prior to initiating the study, ethical approval was obtained from İnönü University Health Sciences Non‐interventional Research Ethics Committee (Number: 2023/4948 and Date: 05.09.2023).

## Conflicts of Interest

The authors declare no conflicts of interest.

## Data Availability

The data that support the findings of this study are available from the corresponding author upon reasonable request.
